# Methods to optimize optical sensing of biotic plant stress – combined effects of hyperspectral imaging at night and spatial binning

**DOI:** 10.1186/s13007-024-01292-2

**Published:** 2024-10-28

**Authors:** Christian Nansen, Patrice J. Savi, Anil Mantri

**Affiliations:** Department of Entomology and Nematology, University of California, UC Davis Briggs Hall, Room 367, Davis, CA 95616 USA

**Keywords:** Hyperspectral imaging, Stress detection, Radiometric repeatability, Reflectance profiling, Circadian rhythm of plants, Image classification

## Abstract

In spatio-temporal plant monitoring, optical sensing (including hyperspectral imaging), is being deployed to, non-invasively, detect and diagnose plant responses to abiotic and biotic stressors. Early and accurate detection and diagnosis of stressors are key objectives. Level of radiometric repeatability of optical sensing data and ability to accurately detect and diagnose biotic stress are inversely correlated. Accordingly, it may be argued that one of the most significant frontiers and challenges regarding widespread adoption of optical sensing in plant research and crop production hinges on methods to maximize radiometric repeatability. In this study, we acquired hyperspectral optical sensing data at noon and midnight from soybean (*Glycine max*) and coleus wizard velvet red *(Solenostemon scutellarioides)* plants with/without experimentally infestation of two-spotted spider mites (*Tetranychus urticae*). We addressed three questions related to optimization of radiometric repeatability: (1) are reflectance-based plant responses affected by time of optical sensing? (2) if so, are plant responses to two-spotted spider mite infestations (biotic stressor) more pronounced at midnight versus at noon? (3) Is detection of biotic stress enhanced by spatial binning (smoothing) of hyperspectral imaging data? Results from this study provide insight into calculations of radiometric repeatability. Results strongly support claims that acquisition of optical sensing data to detect and characterize stress responses by plants to detect biotic stressors should be performed at night. Moreover, the combination of midnight imaging and spatial binning increased classification accuracies with 29% and 31% for soybean and coleus, respectively. Practical implications of these findings are discussed. Study results are relevant to virtually all applications of optical sensing to detect and diagnose abiotic and biotic stress responses by plants in both controlled environments and in outdoor crop production systems.

## Introduction

Optical sensing is being deployed across a wide range of spatio-temporal scales and types of optical sensors to detect and classify reflectance-based plant responses to abiotic and/or biotic stressors. In these applications, it is assumed that [1]: (1) stressors elicit a change in biochemical composition and/or physical structure of plant canopies, and (2) plant canopy changes induced by stressors are linked to detectable and unique leaf reflectance features. A large and growing body of literature supports these coupled assumptions and therefore justify further research and development of systems, in which optical sensing is deployed to detect and diagnose abiotic and biotic stressors of plants [2–5]. However, it is important to highlight that accurate and reliable detection and diagnosis of plant stress hinge on acquisition of strong and consistent optical leaf reflectance features over space and time. In other words, we assume that it is possible to acquire (or perform different types of correction and calibration to obtain) optical sensing data with high level of radiometric repeatability. When the same object is imaged at multiple time points, radiometric repeatability may be calculated as the maximum-minimum range as percentage of average reflectance in a given spectral band, R_x_ [6]:

1 $$\eqalign{& {\rm{Radiometric}}\,{\rm{repeatability }} \cr & {\rm{ = }}\,{\rm{100}}\,{\rm{-}}\,{\rm{(((}}{{\rm{R}}_{\rm{x}}}{\rm{max}}\,{\rm{-}}\,{{\rm{R}}_{\rm{x}}}{\rm{min)}}\,{\rm{ \times }}\,{\rm{100)}} \cr & {\rm{/}}\,{{\rm{R}}_{\rm{x}}}{\rm{average)}} \cr}$$

In optical sensing, radiometric repeatability may be considered an indicator of minimum detection level. Thus, a radiometric repeatability < 95% would suggest that plant stress can only be detected accurately and reliably if causing > 5% change in leaf reflectance. Meaning, level of radiometric repeatability of optical sensing data and ability to accurately detect and diagnose biotic stress are inversely correlated [6–11]. A simple analogy is to consider standard errors on average bars in an ANOVA of two or more treatments. If error bars are large (low repeatability), average treatment responses must be very different to demonstrate statistical significance. On the other hand, small error bars (high repeatability) enable detection of statistically significant treatment effects, even if averages are only marginally different.

**Fig. 1:**
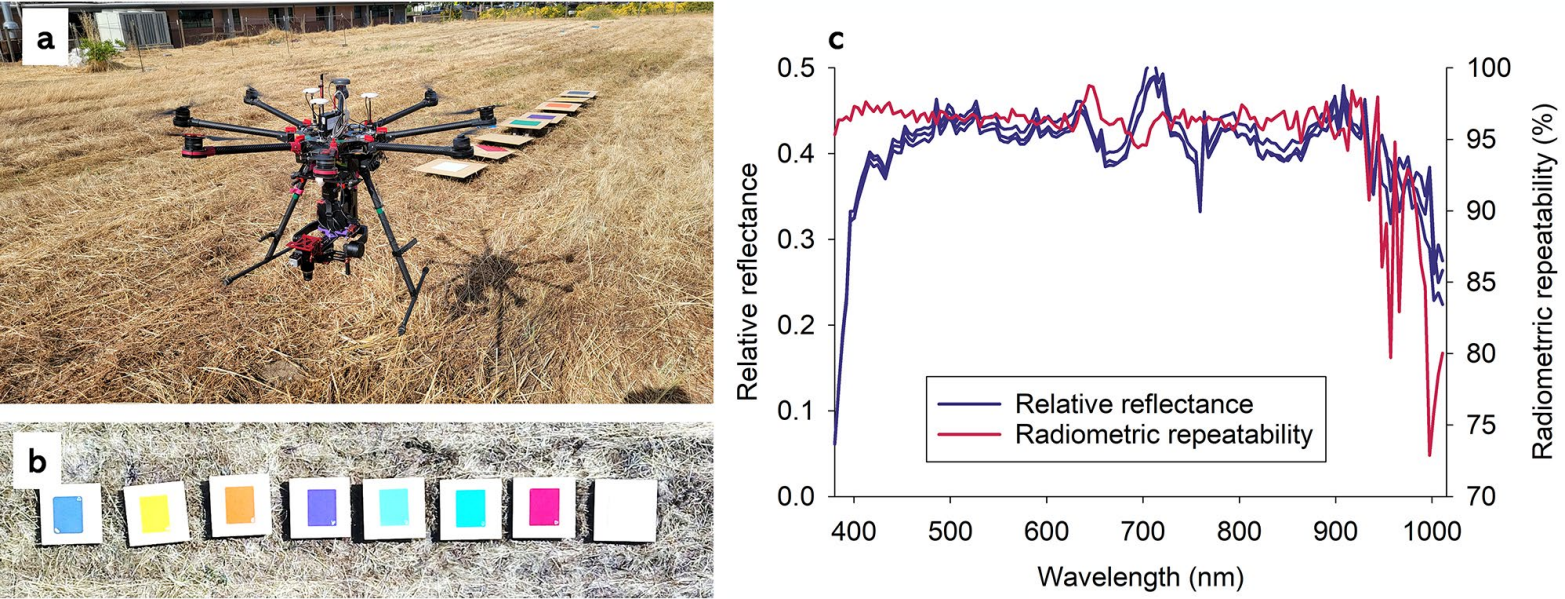
Octocopter drone system (**a**) and color boards (**b**) used to quantify radiometric repeatability. Average reflectance and radiometric repeatability based on three flights within 24 min (**c**)

Factors contributing to low radiometric repeatability of optical sensing data acquired from plants can be broadly divided into four categories: lighting and environment [6, 12–15], imaging systems and optical sensing data sets [16–18], plant agronomics and stressors [19–22], and plant physiology and photoperiods [23–30]. In a few studies, radiometric repeatability was experimentally manipulated by adding known levels of noise to data sets to assess its relative effect on accuracy of classification functions [31, 32]. Radiometric repeatability over time was the focus in a study of optical sensing data acquired from carefully selected target objects (spectrally homogeneous, Lambertian, horizontally placed, and at least 12 × 12 m) [33]. Using ground truthing data from these target objects over a period of nine days, authors showed that radiometric repeatability ranged from 79 to 94% (coefficient of variation ranging from 6 to 21%). A second study involved hyperspectral optical sensing data acquired from colored boards during 52 flight missions on three separate days [6]. As colored boards were assumed not to change in composition and structure during the study, optical sensing data from individual boards were assumed to only vary as a function of lighting and environment. To illustrate and highlight the issue of radiometric repeatability, Fig. 1a shows the drone-based hyperspectral imaging system and the objects being imaged (Fig. 1b) [6]. From this study, Fig. 1c shows the radiometric repeatability of hyperspectral imaging system acquired from white Teflon during three flight missions, each lasting about 1 min: 11:48 am, 11:49 am, and 12:12 pm on the same day.

Within 24 min, and near azimuth on a day with clear blue sky, values in most spectral bands between 380 and 900 nm varied 3–6% (radiometric repeatability = 94–97%), while much lower radiometric repeatability was observed in spectral bands from 900 to 1,015 nm. It seems reasonable to assume that a radiometric repeatability of 94–97% would markedly decrease, if optical sensing data were acquired: across a wider time span within a day, during multiple days, from growing plants, and/or from a large area with topography and scattering from adjacent objects/features. Sensitivity analyses and tests of classification function robustness to radiometric repeatability is a research area, which deserves further attention.

We wish to highlight low radiometric repeatability as a frequently ignored aspect of optical sensing studies. In 2005, K Peleg, GL Anderson and C Yang [34] highlighted the issue of low radiometric repeatability in markedly unambiguous terms: “*Hyperspectral image cubes acquired in consecutive flights over the same target should ideally be identical. In practice*,* two consecutive flights over the same target usually yield significant differences between the image cubes. These differences are due to variations in target characteristics*,* solar illumination*,* atmospheric conditions and errors of the imaging system proper*”. A likely consequence of low radiometric repeatability is that a classification function developed based on a training data set will fail to generate accurate predictions when applied to new and independent optical sensing data. Thus, it may be argued that one of the most significant frontiers and challenges regarding widespread adoption of optical sensing in plant research and crop production hinges on methods to maximize radiometric repeatability.

To maximize radiometric repeatability, this study focused on two distinct but highly complementary methods: (1) acquisition of optical sensing data at night versus during the day, and (2) deployment of spatial binning (pixel binning) of hyperspectral imaging data. If plant stress responses are more pronounced at night, then active (artificial) lighting can be used to provide near-constant lighting during data acquisitions (both over space and time) and therefore mitigate many of the challenges associated with low radiometric repeatability due to lighting and environment. There are several specific research articles and reviews of spatial binning of optical sensing data [35–38]. Spatial binning is the processing step of averaging pixel values, typically in grids of 3 × 3, 4 × 4, 5 × 5, etc., and perceived advantages include reduced effects of outlier pixels, improved signal-to-noise ratio, smaller data sets so that data transfer and classifications are faster, and smoothened optical features. Perceived disadvantages are mainly related to loss of unique optical features due to spatial mixing and loss of spectral features due to spectral binning. In most cases, there will likely be trade-offs, so spatial binning is hypothesized to improve classification accuracies up until a certain point, where averaging of optical features adversely affects classification performance.

**Fig. 2:**
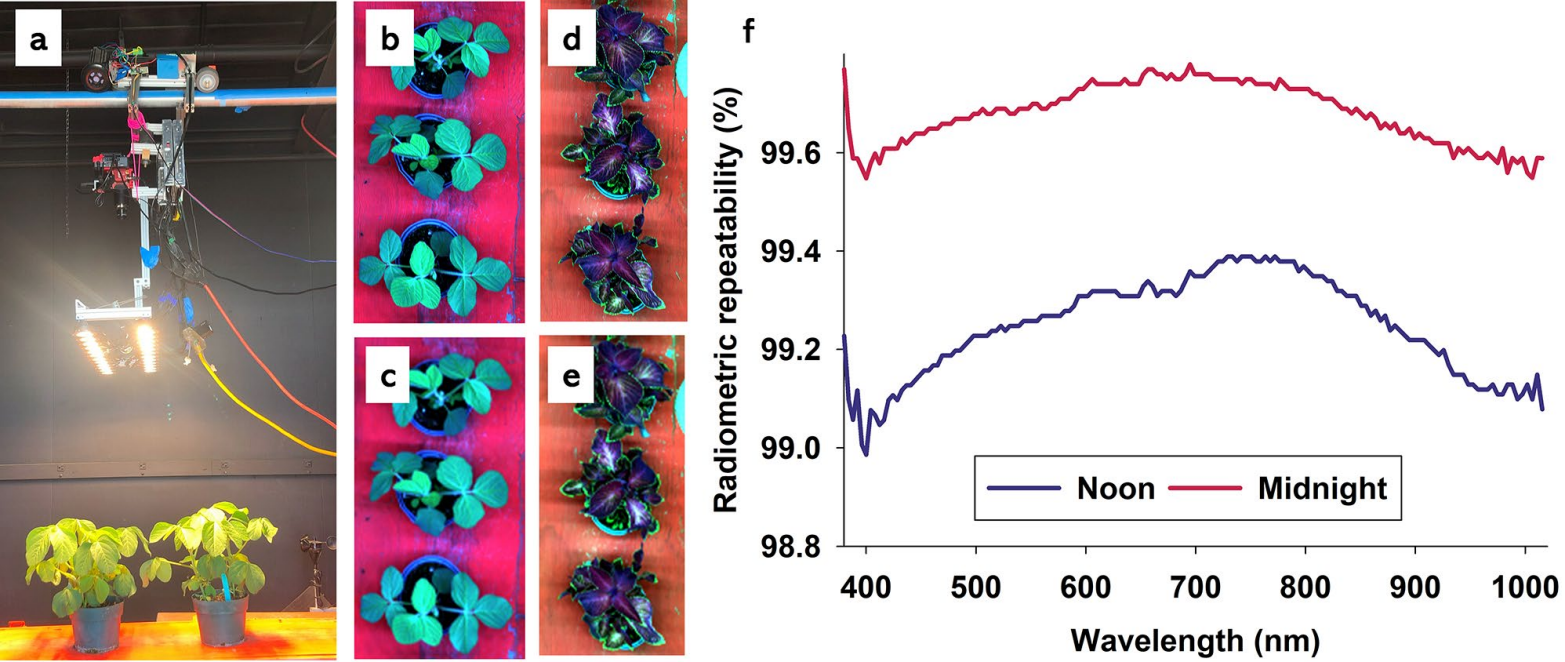
Hyperspectral optical sensing with an active light source inside dark room (**a**). Soybean plants at 1 × 1 (**b**) and 9 × 9 (**c**) spatial binning. Coleus plants at 1 × 1 (**d**) and 9 × 9 (**e**) spatial binning. Radiometric repeatability (%) was calculated (Eq. 1) based on average reflectance of white Teflon on seven days and with data being collected at noon and midnight (**f**)

In this study, we acquired hyperspectral optical sensing data at noon and midnight inside a dark room (Fig. 2a) from soybean (*Glycine max* L.) and coleus wizard velvet red *(Solenostemon scutellarioides* L.) plants with/without experimentally infestation of two-spotted spider mites, (*Tetranychus urticae* Koch). Leaf reflectance from soybean is considered representative for most green plants, while coleus plants have comparatively high concentrations of carotenoids and flavonoids, which are responsible for reddish coloring of leaves [39–42]. Due to different leaf pigment profiles, these two plant species were considered suitable for a comparison, and they are both susceptible to infestations by two-spotted spider mites [43, 44]. Experimental biotic stress levels consisted of no two-spotted spider mite infestation (control), and low and high infestations. The same plants were subjected to optical sensing on seven days during a 16-day period. This enabled characterization of change in leaf reflectance over time. On each of seven days, optical sensing data were acquired at noon and midnight to examine diurnal effects, calculate radiometric repeatability, and to determine whether biotic stress detection accuracy was affected by time of optical sensing. Finally, four levels of spatial binning were performed and their effects on classification accuracies were examined. Results presented here have important practical implications, as: (1) night time optical sensing enables markedly higher ability to control lighting and therefore increase levels of radiometric repeatability, (2) automation of optical sensing can be readily deployed at night time when workers are not present inside greenhouse production systems, and (3) spatial binning can markedly improve data transfer, data classification, and computational complexity needed in high throughput system development. With a focus on maximization of radiometric repeatability, we argue that results presented here are relevant to virtually all optical sensing applications in both controlled environments (with/without artificial lighting) and in outdoor crop production systems.

## Materials and methods

### Plant materials

To avoid unwanted infestations and to treat each plant as a separate experimental unit, individual plants were grown inside screen cages (BugDorm-2120 F insect-rearing tents: width = 60 cm, depth = 60 cm, and height = 60 cm; BioQuip Products). Plants were maintained at controlled greenhouse facilities [25–30 °C (average = 27.8 °C) and 40–50% relative humidity (RH; average = 46.2%)] under natural light conditions (no supplementary lighting). Coleus plugs were transplanted into and soybean seeds planted directly into 6.5-inch pots and continuously supplied fertilization (UC Davis modified Hoagland’s solution) through drip irrigation (ppm): *N* = 131.5, *P* = 40.5, K = 180.0, Ca = 101.0, Mg = 52.0, S = 68.5, Fe = 1.5, Cu = 0.1, Mn = 0.3, Mo = 0.1, and Zn = 0.1. Drip irrigation was delivered to individual pots as two separate irrigation events of 1 min each and 8 h apart (2 × 35 ml = 70 ml per day). For all combinations of crop and treatments, we included eight replicated plants.

### Hyperspectral optical sensing

Optical sensing at noon and midnight was performed inside a dark room immediately adjacent to the greenhouse used to maintain soybean and coleus plants. Each optical sensing event, noon or midnight, was completed within 60 min conditions, so plants were only momentarily outside individual cages. Furthermore, with optical sensing performed inside a dark room with an active halogen light source (Fig. 2a), paired data acquired at noon and midnight were directly comparable, and variation in imaging environments among days was considered negligible. We used a push-broom hyperspectral camera (PIKA L, Resonon Inc., Bozeman, MT, USA) with the following specifications: digital output (12 bit), angular field of view of 7 degrees, objective lens had a 17 mm focal length (maximum aperture of F1.4), spectral range of 380-1,015 nm, and spectral resolution of 150 bands (4.2 nm). Optical sensing data were acquired with a spatial resolution of about 9 pixels mm^− 2^. The hyperspectral camera was mounted on a robotic rail system about 1 m above plants placed on top of a table (Fig. 2a). White Teflon was imaged simultaneously with plants and used as radiometric calibration according to the empirical line method (ELM) [11, 13, 14, 33, 45–51]. Deployment of ELM calibration likely increases levels of radiometric repeatability [6]. Deployment of radiometric filtering [1, 52] was used to only include pixels representing green leaves (exclusion of background).

Hyperspectral imaging was performed on seven days (seven paired combinations of noon and midnight): before infestations (baseline), 7–9 days after infestations (period 1) and 14–16 days (period 2) after infestations. For both plant species, we included three classes: non-infested control plants, low infestation (10 adult two-spotted spider mites per plant) and high infestation (30 adult two-spotted spider mites per plant). With data acquired on seven days × two time points (noon and midnight) × three classes (control, and low and high infestations) × eight plants per treatment, optical sensing data were acquired from 336 combinations of day, time of optical sensing, treatment, and replication for each plant species.

### Data analyses

All data processing, analyses, and classifications were performed in R v3.6.1 (The R Foundation for Statistical Computing, Vienna, Austria).

#### Diurnal variation of leaf reflectance

We examined paired average reflectance profiles from the seven time points at noon and midnight. This analysis was based exclusively on optical sensing data from control plants, and data from each plant species were analyzed separately. A looped paired t-test was used to examine reflectance values in each of the 150 spectral bands. For each of the two plant species, the main purpose was to identify spectral regions with high sensitivity to growth of plants over time. Average reflectance profiles from white Teflon and from control plants were also used to calculate radiometric repeatability (based on Eq. 1).

#### Diurnal variation of biotic stress response

We performed the same looped paired t-test, as described above, to compare control plants with those subjected to high infestation (low infestation plants were excluded from this analysis).

#### Importance of spatial binning

We performed SVM classification [using the library(e1071) with linear kernel function and no specific hyperparameters (i.e., cost or gamma)], and optical sensing data were divided into four classes: baseline, non-infested control, and low and high infestations. Separate SVM classifications were performed for optical sensing data acquired at noon and at midnight. SVM classifications were performed based on grouped optical sensing data acquired 7–9 days after infestations (period 1) and 14–16 days after infestations (period 2). Thus, one SVM classification included baseline data and data from period 1, while a second SVM classification included baseline data and data from period 2. This allowed us to determine relative changes over time, as baseline data were predicted to be classified with higher accuracy, when combined with data from period 2 than when combined with data from period 1. Furthermore, we conducted SVM classifications based on four spatial binning levels: no spatial binning (using individual pixels), 5 × 5 (25-fold data reduction), 7 × 7 (49-fold data reduction), and 9 × 9 (81-fold data reduction). Accordingly, a total of 32 SVM classifications were performed [two plant species × two periods × two times of day (noon and midnight) × four levels of spatial binning]. In all SVM classifications, irrespectively of level of spatial binning, it was ensured that numbers of observations in classes were balanced. This is important, as classifications based on SVM and other functions are generally sensitive to data balance among classes [53, 54]. Representative photos of original data (no spatial binning) and 9 × 9 spatial binning are presented in Fig. 2b-e. As assessment of classification performances, we generated Kappa values [55] and also included 10-fold cross-validation [56–58]. Regarding interpretation of Kappa values, the following is generally accepted [55]: 0 = poor, 0.01– 0.20 = slight, 0.21–0.40 = fair, 0.41–0.60 = moderate, 0.61–0.80 = substantial, and 0.81–1.00 = almost perfect. In k-fold cross-validation, training data sets are divided into ‘k’ equal portions, which in this study was set at 10 (so 18 observations in each portion). Classification models were trained on ‘k-1’ of these portions, while a remaining portion is used for validation. This process was repeated ‘k’ times, with each fold serving as validation, and results from ‘k’ tests are averaged to produce a single estimation of model classification performance.

## Results and discussion

### Temporal trends of optical sensing data

**Fig. 3:**
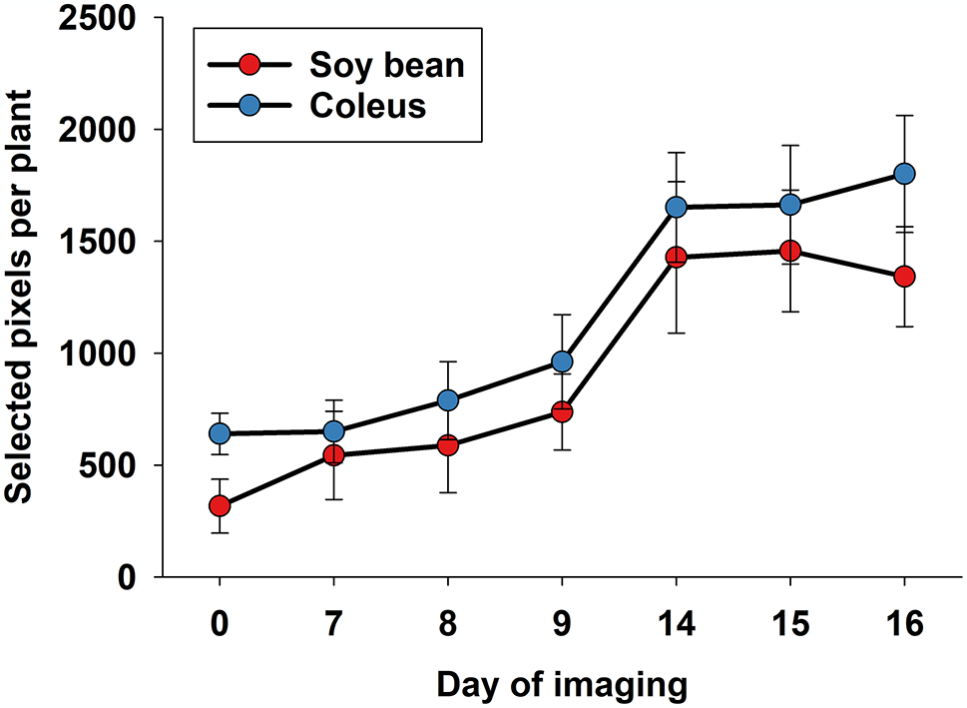
Average number of green pixels per plant was calculated and used as indicator of plant growth during the course of the study

Figure 3 shows average numbers of green pixels for each plant species over time, and it is seen that bean plants grew about 4-fold, while coleus plants grew about 3-fold. We highlight this temporal variation in size (average number of green pixels per plant), because it can lead to unbalanced data sets and therefore biased statistical outcomes [59], if disproportionally more pixels are included from larger plants. To avoid concerns about unbalanced data, all statistical analyses were based on randomly selected but fixed numbers of pixels (also when spatial binning was deployed) from each combination of time points and treatment. This issue of unbalanced data sets due to growth of plants has broad relevance, especially if treatments (such as drought or fertilizer) directly impact plant growth, and/or if optical sensing data are acquired over time.

### Diurnal variation of optical sensing data

**Fig. 4:**
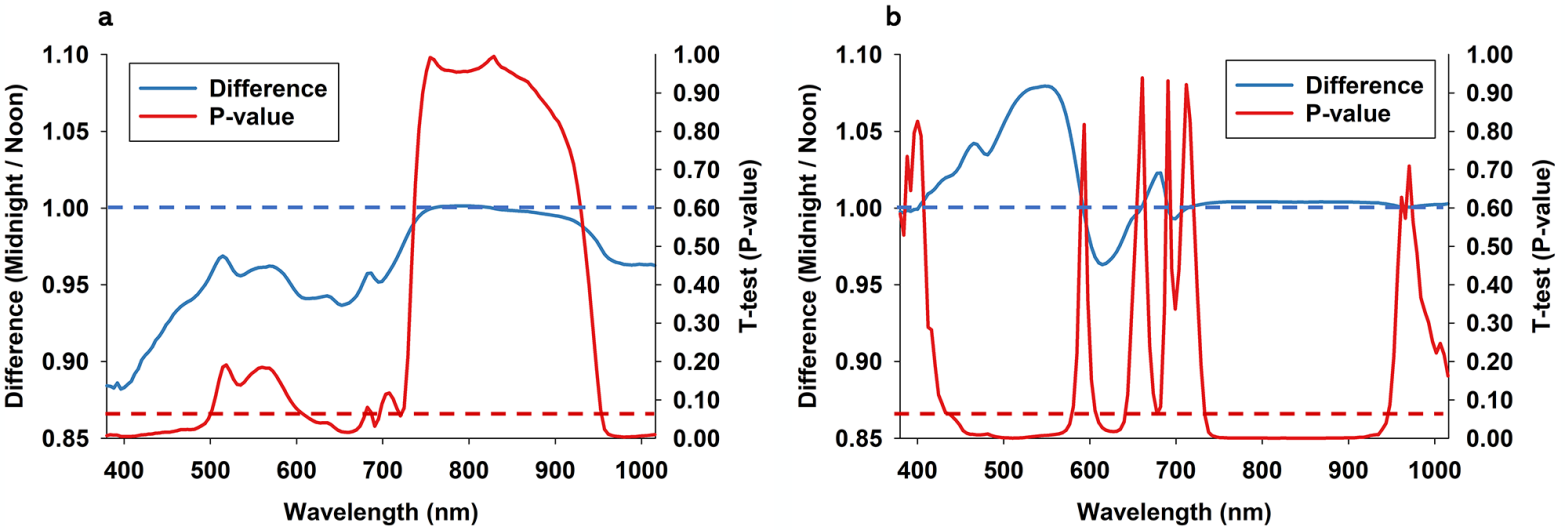
Average effect of time of optical sensing is illustrated as a ratio (midnight/noon) for soybean (**a**) and coleus (**b**). Horizontal blue dotted line = 1, which equals no effect of time of optical sensing. For all 150 spectral bands from 380-1,015, we performed paired t-tests, and p-values are presented as continuous red line. Horizontal red dotted denotes significance at the 0.05-level

Average effect of time of optical sensing was calculated as the relative difference (midnight/noon) in all 150 spectral bands (Fig. 4). Thus, horizontal blue dotted lines denote a ratio = 1, which implies no difference between noon and midnight. A ratio > 1 suggests higher leaf reflectance at midnight, while a ratio < 1 suggests that leaves were darker at night compared to at noon. Horizontal red dotted lines denote paired t-test p-values = 0.05, so that spectral bands below this threshold suggest statistical significance. Regarding soybean (Fig. 4a), midnight leaf reflectance was lower in all spectral bands, except for spectral bands from 730 to 900 nm. It is also seen that leaf reflectance at midnight was significantly lower in spectral bands from 380 to 500 nm, 600–700 nm, and 960-1,015 nm. Accordingly, these spectral regions were considered spectral regions with strongest responses to time of optical sensing by soybean plants. These spectral ranges align partially with those identified as possible indicators of chlorophyll a and b [60–63]. Reflectance near 700 nm has been shown experimentally to be associated with chlorophyll a content [60, 64, 65]. Based on readings in 15-min intervals on three separate days, the red portion of the radiometric spectrum (600–700 nm) of soybean plants has been shown to vary as much as 140% [66].

Regarding coleus (Fig. 4b), leaf reflectance was generally higher at midnight than at noon, except for spectral bands from 580 to 670 nm. There was a prominent leaf reflectance peak in spectral bands from 400 to 600 nm. Examination of Fig. 4b suggests negligible relative difference in spectral bands from 750 to 950 nm (close to value = 1). However, paired t-test results of reflectance values in these spectral bands suggested highly significant responses to time of optical sensing. Furthermore, it highlights that visual interpretations of average responses may be slightly deceiving and should be accompanied by statistical analyses. In addition, leaf reflectance at midnight was significantly higher in spectral bands from 450 to 580 nm, and significantly lower in spectral bands from 600 to 630 nm. These spectral ranges align partially with those identified as possible indicators of carotenoids and anthocyanins [62, 67, 68]. As coleus species typically have high anthocyanin content [40], it is not surprising that these plants responded differently in terms of leaf reflectance to time of optical sensing compared to soybean plants.

Summarizing methods and results: we randomly selected 1,200 pixels for each combination of plant species, day of imaging, and time of optical sensing. Average reflectance in 150 individual spectral bands were compared using a paired t-test, based on seven pairs of values in each spectral band (noon versus midnight). From these analyses, we conclude that the two plant species responded differently to time of optical sensing, as coleus plants were generally brighter at midnight, while soybean plants became darker compared to at noon. In addition, the two plant species showed significant responses in different spectral regions. However, for both plant species we conclude that time of optical sensing has a profound influence on optical sensing data. Importantly, magnitude of average difference was not a reliable indicator of statistical significance. Moreover, Fig. 4b shows only a modest increase in leaf reflectance in spectral bands from 740 to 940 nm, but statistical comparisons were highly significant. Thus, this spectral region is interpreted as possessing a high level of radiometric repeatability. Conversely, Fig. 4a showed a considerable decrease in leaf reflectance in spectral bands from 500 to 600 nm, but differences in individual bands were not statistically significant. Thus, this spectral region was likely associated with low radiometric repeatability. This exercise highlights the importance of assessing relative responses based on statistical comparisons rather than qualitative/visual interpretations of treatment responses in individual spectral bands. Furthermore, it provided valuable insight into spectral regions with strong response to photoperiod.

Actual impact of physiological dynamics on leaf reflectance has, to the best of our knowledge, not been thoroughly examined, but such knowledge may provide insight into possible factors adversely affecting radiometric repeatability of optical sensing data from plants. Reviews of spectral indices show that spectral bands from 900 to 970 nm are frequently used [3, 69–73]. With spectral bands in this region showing a highly significant response to noon versus midnight in coleus, our results suggest that such spectral indices should be used with caution in optical sensing of plants with high concentrations of anthocyanins.

### Diurnal variation of radiometric repeatability

**Fig. 5:**
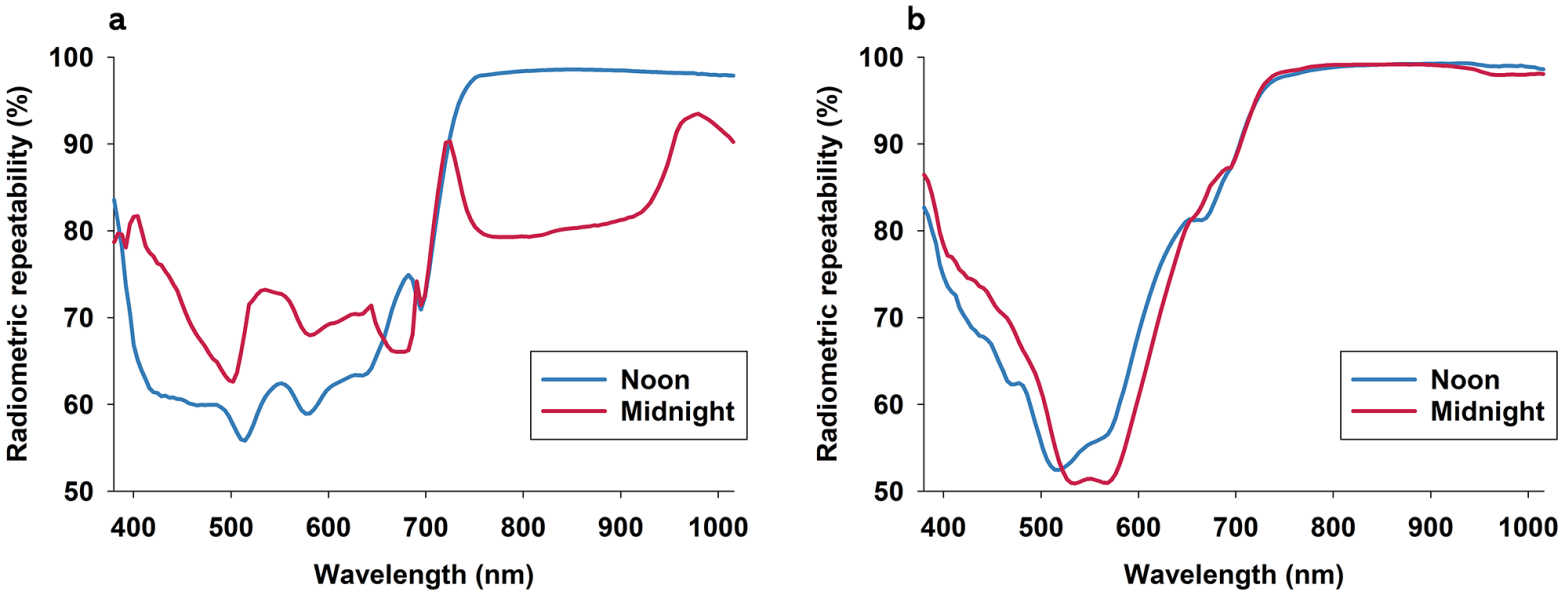
Radiometric repeatability of average reflectance profiles from soybean (**a**) and coleus (**b**) control plants was calculated based on Eq. 1

Application of Eq. 1 to average reflectance data acquired from white Teflon was used to calculate radiometric repeatability of imaging system and imaging conditions (Fig. 2f). We found that radiometric repeatability exceeded 99% in all spectral bands, and it was consistently higher when hyperspectral imaging data were acquired at midnight. This analysis is rarely included in published optical sensing studies, but it markedly improves ability to interpret optical trends and plant responses to treatments. Moreover, with demonstration of < 1% stochastic noise in reflectance data, we are in position to make much stronger statements about plant responses to photoperiod and to a biotic stressor. Regarding soybean (Fig. 5a), radiometric repeatability was below 80% in spectral bands from 380 to 700 nm, and it was lowest when hyperspectral imaging data were acquired at noon. In spectral bands from about 740-1,015 nm, radiometric repeatability was near 100% at noon but considerably lower when hyperspectral imaging data were acquired at midnight. Regarding coleus (Fig. 5b), radiometric repeatability was generally low (< 80%) in spectral bands from 400 to 650 nm, while it was near 100% in spectral bands from about 740-1,015 nm, irrespectively of whether hyperspectral imaging data were acquired at noon or midnight.

We wish to re-emphasize that hyperspectral imaging data were acquired inside a dark room with artificial lighting, and that ELM calibration was performed for each time point. Consequently, low/reduction of radiometric repeatability is considered to be exclusively attributed to physiological changes in response to photoperiod. As part of interpreting results presented in Fig. 5, it is paramount to highlight that reflectance profiles used to calculate radiometric repeatability represented averages of thousands of pixels (see Fig. 3), as hundreds of pixels were selected from each of eight replicated plants. Thus, reductions of radiometric repeatability of 20% or more represented marked changes in leaf reflectance. Obvious consequences of results presented in Fig. 5 are: (1) stress signals representing < 40% change in leaf reflectance may not be detectable, if based on spectral bands from 380 to 700 nm, (2) if using spectral bands from 380 to 700 nm, then acquisition of hyperspectral imaging data at midnight provide higher likelihood of accurate crop stress detection, 4) spectral bands from 730 to 1,015 nm were associated with very high radiometric repeatability in both crops, and irrespectively of whether hyperspectral imaging data were acquired at noon or midnight, and 4) radiometric repeatability of crops with different pigment profiles appear to show high degree of variability in terms of responses to time of day of hyperspectral imaging.

The observed optical trends presented in Fig. 5 are likely due to complex physiological plants responses to photoperiod [30]. Concentration of chlorophyll has been shown to follow diurnal fluctuations in a number of plants, including tomato [24], petunia [27], tobacco [26], and wheat [23, 74]. Anthocyanins also show diurnal fluctuations [40]. At a high spatial resolution, M Busheva, G Garab, E Liker, Z Tóth, M Szèll and F Nagy [23] showed that contents of chlorophyll pigments a and b in wheat leaves (basal, mid, and tip) changed markedly, both diurnally and seasonally. SJ Britz and WR Briggs [75] studied an Ulva species and showed that chloroplasts were near the outer leaf surface during the day, while at night, these pigments were mainly located along the leaf sides, and the absorbance of radiometric energy was low. This spatio-temporal variability and mobility of chloroplasts and concentrations of pigments underscore that plants have mechanisms to regulate (and possibly optimize) their investment in photosynthesis. From experimental studies of green leaves, it has been demonstrated that concentrations of plant pigments can be quantified non-destructively based on wavelength-specific leaf reflectance features (“R” denotes relative reflectance, “ρ” denotes reciprocal reflectance) [60–63, 67, 68], including: chlorophyll a (R700, R750/R550, and R750/R700, ρ710- ρ790), chlorophyll b (R672/R550, R860/R550), carotenoids (R510, R531, R550, R700), anthocyanins (ρ550- ρ710). Thus, under assumption of diurnal variations in pigment concentrations in plants, it would be expected that leaf reflectance acquired during the day and at night vary in some of these specific wavelengths. However, we are unaware of studies experimentally testing whether such diurnal dynamics of pigments and plant physiology may influence magnitude and types of reflectance responses to biotic stressors.

### Diurnal variation of biotic stress response

**Fig. 6:**
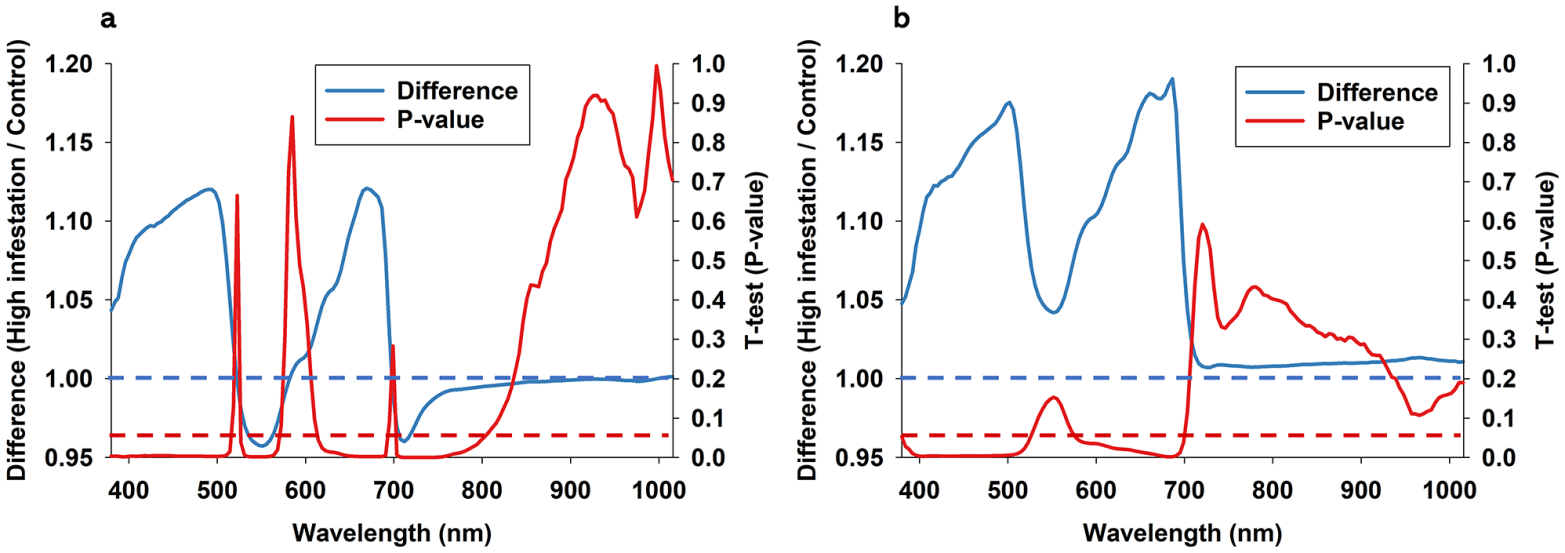
Average difference between plants subjected to high two-spotted spider mite infestations and control plants is illustrated as a ratio (high infestation/ control) for soybean when imaged at noon (**a**) or at midnight (**b**). Horizontal blue dotted line = 1, which equals no effect of time of optical sensing. For all 150 spectral bands from 380-1,015, we performed paired t-tests, and p-values are presented as continuous red line. Horizontal red dotted denotes significance at the 0.05-level

In a first analysis of stress response, only control and high infestation plants (not low infestation) were compared and subjected to the same analytical approach as used to examine leaf reflectance responses to time of optical sensing. Regarding soybean at noon (Fig. 6a), it is seen that two-spotted spider mite infestations elicited a significant increase in leaf reflectance in spectral bands from 380 to 510 nm and 570–700 nm, while infestations caused a significant decrease in leaf reflectance in spectral bands from 700 to 800 nm. A similar, but stronger, stress response was observed in optical sensing data acquired from soybean at midnight with highly significant increases in leaf reflectance in spectral bands from 380 to 530 nm and 570–700 nm (Fig. 6b).

**Fig. 7:**
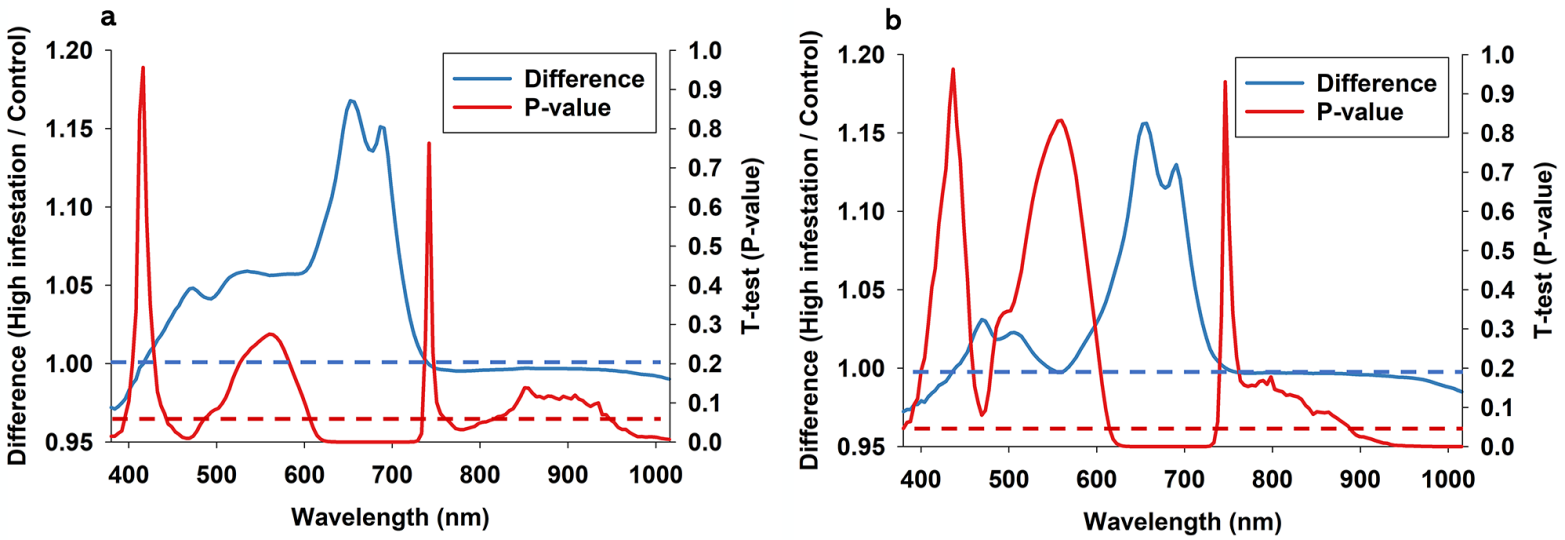
Average difference between plants subjected to high two-spotted spider mite infestations and control plants is illustrated as a ratio (high infestation/ control) for coleus when imaged at noon (**a**) or at midnight (**b**). Horizontal blue dotted line = 1, which equals no effect of time of optical sensing. For all 150 spectral bands from 380-1,015, we performed paired t-tests, and p-values are presented as continuous red line. Horizontal red dotted denotes significance at the 0.05-level

Regarding coleus at noon (Fig. 7a), it is seen that two-spotted spider mite infestations elicited a significant increase in leaf reflectance in spectral bands from 450 to 480 nm and 615–720 nm, while infestations caused a significant decrease in leaf reflectance in spectral bands near 780 nm and from 950 to 1,015 nm. Regarding optical sensing data acquired from coleus at midnight (Fig. 7b), two-spotted spider mite infestations elicited a significant increase in spectral bands from 615 to 720 nm and a small but significant decrease in spectral bands from 890 to 1,015 nm. Several studies have described increase in leaf reflectance in response to two-spotted spider mite infestation [1, 76–82].

**Table 1:** SVM classifications of optical sensing data from soybean

Binning	Pixels	Diurnal	Period	Base	Control	Low	High	K-1	Kappa
1 by 1	1200	Noon	1	0.87	0.65	0.62	0.62	63.04	0.59
5 by 5	1200	Noon	1	0.97	0.77	0.68	0.70	75.58	0.71
7 by 7	600	Noon	1	0.98	0.79	0.70	0.70	75.96	0.72
9 by 9	375	Noon	1	0.98	0.79	0.71	0.78	76.93	0.76
1 by 1	1200	Noon	2	0.99	0.69	0.52	0.65	65.79	0.62
5 by 5	1200	Noon	2	1.00	0.71	0.61	0.75	73.52	0.69
7 by 7	600	Noon	2	1.00	0.76	0.58	0.78	74.58	0.71
9 by 9	375	Noon	2	1.00	0.73	0.69	0.78	75.80	0.73
1 by 1	1200	Midnight	1	0.97	0.71	0.65	0.62	68.56	0.65
5 by 5	1200	Midnight	1	0.99	0.86	0.76	0.77	82.67	0.79
7 by 7	600	Midnight	1	0.99	0.85	0.75	0.79	83.46	0.80
9 by 9	375	Midnight	1	0.99	0.88	0.78	0.77	83.60	0.81
1 by 1	1200	Midnight	2	0.99	0.75	0.58	0.64	68.67	0.66
5 by 5	1200	Midnight	2	1.00	0.84	0.60	0.74	77.17	0.73
7 by 7	600	Midnight	2	1.00	0.86	0.63	0.78	78.58	0.76
9 by 9	375	Midnight	2	1.00	0.85	0.57	0.81	77.27	0.74
		Noon		0.97a	**0.74a**	0.64a	0.72a	72.65a	0.69a
		Midnight		0.99a	**0.82b**	0.67a	0.74a	77.50a	0.74a

“Binning” denotes level of spatial binning (averaging of pixels). “Pixels” denotes numbers of pixels randomly selected for each of the four categories. This number of pixels from each category was fixed for each analysis to ensure balance of data. “Period”: 1 = 7–9 days after infestations (Period 1) and 14–16 days (Period 2) after infestations with two-spotted spider mites. Optical sensing data in four categories (Base = baseline, Control = non-infested plants, Low = low infestation, and High = high infestation). K-1 denotes k-fold cross-validation and Kappa = Kappa value. Average values for noon and midnight were compared based on one-way anova and letters denote statistical difference at the 0.05-level

In a second analysis, we conducted SVM classification of plants divided into four classes: baseline, control, and low and high infestations. Table 1 shows outcomes from classifications of soybean optical sensing data acquired at noon and midnight and in response to four levels of spatial binning. At progressively higher levels of spatial binning, available numbers of pixels became a limitation, so different fixed number of pixels were used for different levels of spectral binning. For all four classes (baseline, control, and low and high infestations), and also regarding overall accuracy, classification accuracies were numerically highest at midnight compared to noon, but only control plants showed significantly higher accuracy (*P* < 0.05). The class, baseline, was classified with highest level of accuracy, especially for period 2. As baseline data were acquired about two weeks prior to period 2, this result was expected. Furthermore, it highlights the important fact that leaf reflectance changes during time periods of a few days. Low infestations were associated with lowest classification accuracies.

**Table 2:** SVM classifications of optical sensing data from coleus

Binning	Pixels	Diurnal	Period	Base	Control	Low	High	K-1	Kappa
1 by 1	1200	Noon	1	0.80	0.60	0.42	0.50	51.83	0.44
5 by 5	1200	Noon	1	0.90	0.60	0.47	0.68	62.38	0.55
7 by 7	600	Noon	1	0.91	0.64	0.54	0.69	65.13	0.59
9 by 9	375	Noon	1	0.93	0.59	0.55	0.66	63.20	0.58
1 by 1	1200	Noon	2	0.89	0.63	0.47	0.59	57.81	0.52
5 by 5	1200	Noon	2	0.96	0.62	0.51	0.68	65.98	0.59
7 by 7	600	Noon	2	0.96	0.63	0.54	0.66	64.83	0.59
9 by 9	375	Noon	2	0.96	0.66	0.52	0.69	65.73	0.61
1 by 1	1200	Midnight	1	0.77	0.60	0.36	0.58	51.67	0.44
5 by 5	1200	Midnight	1	0.90	0.67	0.49	0.69	64.83	0.58
7 by 7	600	Midnight	1	0.91	0.71	0.48	0.73	65.33	0.61
9 by 9	375	Midnight	1	0.89	0.71	0.54	0.75	67.13	0.63
1 by 1	1200	Midnight	2	0.88	0.62	0.40	0.58	55.60	0.49
5 by 5	1200	Midnight	2	0.95	0.66	0.51	0.71	67.90	0.62
7 by 7	600	Midnight	2	0.95	0.67	0.52	0.74	66.29	0.63
9 by 9	375	Midnight	2	0.97	0.69	0.52	0.72	67.13	0.64
		Noon		0.91	0.62	0.50	0.64	62.11	0.56
		Midnight		0.90	0.67	0.47	0.69	63.24	0.58

“Binning” denotes level of spatial binning (averaging of pixels). “Pixels” denotes numbers of pixels randomly selected for each of the four categories. This number of pixels from each category was fixed for each analysis to ensure balance of data. “Period”: 1 = 7–9 days after infestations (Period 1) and 14–16 days (Period 2) after infestations with two-spotted spider mites. Optical sensing data in four categories (Base = baseline, Control = non-infested plants, Low = low infestation, and High = high infestation). K-1 denotes k-fold cross-validation and Kappa = Kappa value

This was to be expected, as optical data from these plants would likely be misclassified as either control or high infestation. For all four combinations of time of optical sensing (noon and midnight) and time period (1 and 2), there was an increase in Kappa values as a function of spatial binning. Regarding interpretation of Kappa values from soybean classifications, most values were within the 0.61–0.80 range, which is considered “substantial” [55]. Table 2 shows outcomes from classifications of coleus optical sensing data acquired at noon and midnight and in response to four levels of spatial binning. Two classes (control, and high infestations) as well as overall accuracy (K-1) and average Kappa values were numerically highest at midnight compared to noon, but none of them showed significantly higher accuracy (*P* > 0.05). Regarding interpretation of Kappa values from coleus classifications, most values were within the 0.41–0.60 range, which is considered “moderate” [55]. As it was seen in analyses of soybean: (1) There was a positive correlation between level of spatial binning and accuracy of classifications of optical sensing data acquired from coleus. (2) Baseline was classified with a high level of accuracy, especially for period 2. (3) Low infestations were associated with lowest classification accuracies.

In summary, classification results based on optical sensing data from both soybean and coleus showed that: (1) accuracies were numerically higher when plants were imaged at midnight, (2) spatial binning increased accuracies, and (3) temporal comparison (comparing baseline data with data from periods 1 and 2, respectively) showed clear trends of leaf reflectance changing as a function of plant growth over time. These trends were observed based on balanced data (fixed numbers of pixels included from all plant categories), so they cannot be interpreted as possible artefacts and/or flaws in data structures.

## Final perspectives

Optical sensing, with hyperspectral imagers and other sensors, is a crucially important method to monitor and manage crops in agriculture and plants in non-agricultural environments [11, 13, 83, 84]. Thus, it is paramount that research addresses and finds solutions to the most challenging bottlenecks restricting further adoption of optical sensing. We argue that methods to maximize radiometric repeatability of optical sensing data should be regarded as a primary research priority and frontier. Based on average reflectance profiles acquired from control plants on seven separate days under controlled/artificial lighting and with ELM calibration, we demonstrated radiometric repeatability varies considerably among spectral bands: low radiometric repeatability in range from 380 to 700 nm, and high radiometric repeatability in range from 720 to 1,015 nm. With radiometric repeatability falling below 80% in some spectral regions, results from this study corroborate concerns raised and results presented almost 25 years ago [34]. Facing challenges with low radiometric repeatability, we tested relative effects of time of optical sensing of hyperspectral imaging and of spatial binning on classification accuracies. As seen in Table 1, classification of optical sensing from soybean plants at noon with no spatial binning was associated with Kappa values of 0.59 (period 1) and 0.62 (period 2), while midnight Kappa values were 0.81 (period 1) and 0.74 (period 2). Thus, the combination of midnight imaging and spatial binning increased classification accuracies by about 29% (from 0.60 to 0.77). Classification of optical sensing from coleus plants at noon with no spatial binning was associated with Kappa values of 0.44 (period 1) and 0.52 (period 2), while midnight Kappa values were 0.63 (period 1) and 0.64 (period 2) (Table 2). Thus, the combination of midnight imaging and spatial binning increased classification accuracies by about 31% (from 0.48 to 0.63).

Optical sensing at midnight with active light source would mitigate a number of challenges, including radiometric calibration. ELM calibration is probably the most commonly used method of radiometric calibration of (airborne) optical sensing data [14], and it has been thoroughly reviewed [11, 47, 51]. Other radiometric calibration methods are based on quantification of “true reflectance” as a reference [7, 14, 34, 66]. ELM calibration requires placement and retrieval of reference boards, which must be placed sufficiently frequent to account for temporal variations in atmospheric conditions and sun parameters. Placement and retrieval of calibration boards are time and labor consuming, and they must be placed in ways that minimize radiometric noise due to projection angle issues and/or shadows being cast by adjacent objects. Furthermore, calibration boards must be kept clean, stored properly, and high-quality calibration boards are often costly. Additionally, continuous use of commercially available calibration boards (based on supplier and material) under field conditions may lead to change in their optical characteristics over time [84]. These challenges associated with ELM-based radiometric calibration are highlighted, because they contribute significantly to risks of low radiometric repeatability. And most of concerns with ELM-based radiometric calibration may be mitigated by opting for midnight optical sensing with an active and constant light source. In open field cropping systems, midnight optical sensing may represent some logistical challenges. However, automated rovers or pivot irrigation could be outfitted with active light sources and optical sensor, and if rovers are deployed swarms, then data from large crop areas could be acquired in a timely fashion. Costs of optical sensing equipment could be a significant constraint, but it may be justified if radiometric repeatability of optical sensing data can be markedly improved. In controlled environments, optical sensing at midnight would likely be associated with less (negligible) concerns about fluctuating lighting conditions and/or concerns about beams and other structural features casting shadows and scattering. Furthermore, optical sensing would be performed when workers are not present, so there would be no down-time (loss of productivity). Optical sensing at night would also benefit from another critically important aspect, which is that chloroplasts tend to be more stable in the dark. Moreover in darkness, chloroplasts are not subjected to photooxidative stress, which is a major cause of chloroplast damage induced by light conditions [30]. Results from this study provided strong support for claims that acquisition of optical sensing data to detect and characterize biotic stress responses by plants should be performed at night. Additionally, we demonstrated marked benefits of deploying spatial binning.

## Data Availability

Optical sensing data supporting this study are in BIP format and are available from the authors upon reasonable request and with permission.
